# Cellular and behavioral effects of lipopolysaccharide treatment are dependent upon neurokinin-1 receptor activation

**DOI:** 10.1186/s12974-018-1098-4

**Published:** 2018-02-27

**Authors:** Hannah D. Fulenwider, Britessia M. Smith, Anna S. Nichenko, Jessica M. Carpenter, Sadie E. Nennig, Kejun Cheng, Kenner C. Rice, Jesse R. Schank

**Affiliations:** 1 Department of Physiology and Pharmacology, College of Veterinary Medicine, University of Georgia, 501 D.W. Brooks Drive, Athens, GA 30602 USA; 2 Department of Kinesiology, University of Georgia, 115 Ramsey Center, 330 River Road, Athens, GA 30602 USA; 3 NIDA and NIAAA IRP, 9800 Medical Center Drive, Rm 228A MSC-3373, Bethesda, MD 20892 USA; 4 Present Address: Office of Pharmaceutical Quality, Center for Drug Evaluation and Research, Food and Drug Administration, Silver Spring, MD USA

**Keywords:** Neurokinin-1 receptor, Nuclear factor kappa B, Lipopolysaccharide, Inflammation, Cytokines, Anhedonia, Depression, Neuroimmune system

## Abstract

**Background:**

Several psychiatric conditions are affected by neuroinflammation and neuroimmune activation. The transcription factor nuclear factor kappa light-chain-enhancer of activated B cells (NFkB) plays a major role in inflammation and innate immunity. The neurokinin-1 receptor (NK1R) is the primary endogenous target of the neuroactive peptide substance P, and some data suggests that NK1R stimulation may influence NFkB activity. Both NK1R and NFkB have been shown to play a functional role in complex behaviors including stress responsivity, depression, and addiction. In this study, we test whether NFkB activity in the brain (stimulated by lipopolysaccharide administration) is dependent upon the NK1R.

**Methods:**

Adult male Wistar rats were treated systemically with the NK1R antagonist L822429 followed by administration of systemic lipopolysaccharide (LPS, a strong activator of NFkB). Hippocampal extracts were used to assess expression of proinflammatory cytokines and NFkB-DNA-binding potential. For behavioral studies, rats were trained to consume 1% (*w*/*v*) sucrose solution in a continuous access two-bottle choice model. After establishment of baseline, animals were treated with L822429 and LPS and sucrose preference was measured 12 h post-treatment.

**Results:**

Systemic LPS treatment causes a significant increase in proinflammatory cytokine expression and NFkB-DNA-binding activity within the hippocampus. These increases are attenuated by systemic pretreatment with the NK1R antagonist L822429. Systemic LPS treatment also led to the development of anhedonic-like behavior, evidenced by decreased sucrose intake in the sucrose preference test. This behavior was significantly attenuated by systemic pretreatment with the NK1R antagonist L822429.

**Conclusions:**

Systemic LPS treatment induced significant increases in NFkB activity, evidenced by increased NFkB-DNA binding and by increased proinflammatory cytokine expression in the hippocampus. LPS also induced anhedonic-like behavior. Both the molecular and behavioral effects of LPS treatment were significantly attenuated by systemic NK1R antagonism, suggesting that NK1R stimulation lies upstream of NFkB activation following systemic LPS administration and is at least in part responsible for NFkB activation.

## Background

Discovered by Sen and Baltimore in 1986, nuclear factor kappa light-chain-enhancer of activated B cells (NFkB) is a widely expressed transcription factor known for its involvement in inflammation and the innate immune system [1–3]. NFkB is activated after exposure to a variety of stimuli, such as oxidative stressors or pathogens, and induces the expression of genes involved in the innate immune response [4]. For example, systemic treatment with lipopolysaccharide (LPS), a bacterial endotoxin, leads to significant increases in NFkB activity and proinflammatory cytokine production [5]. NFkB activation and subsequent cytokine production have also been shown to influence depressive-like behaviors, specifically social avoidance and anhedonia [6–11]. Human studies have also demonstrated that NFkB activation by low-dose LPS injection induces anhedonia, as evidenced by decreased striatal activity during a reward-related task, and LPS-exposed subjects also reported depressed mood [12]. Additional studies have also found that stress-induced anhedonia and decreased hippocampal neurogenesis are dependent on the proinflammatory cytokine interleukin-1β (IL-1β) and NFkB activation [13].

The neurokinin-1 receptor (NK1R) is the primary endogenous target of the neuropeptide substance P (SP). SP and the NK1R are widely expressed throughout the central nervous system (CNS) and, like NFkB, are involved in stress, anxiety, and depression [14–20]. The NK1R has also been found to play a prominent role in inflammatory responses within the CNS and in the periphery via a process known as neurogenic inflammation [21, 22]. NK1Rs are expressed by glial cells, and their stimulation by SP can enhance the expression and release of inflammatory cytokines [23–25]. NK1R inhibition, either by genetic deletion or pharmacological antagonism, also attenuates CNS inflammation in response to bacterial infection [26–28].

The NK1R and NFkB have been found to interact directly. For example, NK1R stimulation leads to increased NFkB activation [24, 25, 29]. Treatment with SP causes increased NFkB activation and proinflammatory cytokine production, which is attenuated by selective NK1R antagonism [30, 31]. This relationship may be bidirectional, as it is known that the TACR1 gene (gene for NK1R) contains NFkB responsive elements in its promoter [32]. In regard to LPS treatment specifically, NK1R antagonist treatment prevents cytokine release and the detrimental effects of LPS exposure in the lung and liver [31, 33]. Additionally, the central SP/NK1R system mediates the fever response to peripheral LPS administration [34, 35]. However, the role of the NK1R specifically in LPS-induced cytokine expression and NFkB activation within the CNS, and the resulting behavioral consequences, has not been assessed in an in vivo model.

The current study aims to characterize the cellular relationship between the NK1R and NFkB systems in the brain, as well as resulting effects on behavior, specifically, motivated behavior that drives sucrose consumption. We hypothesized that systemic antagonism of the NK1R prior to LPS administration would prevent the LPS-induced increase in NFkB activity, proinflammatory cytokine expression, and anhedonic behavior.

## Methods

### Animals

All experiments were conducted using adult male Wistar rats (Charles River Laboratories, 175–200 g at time of arrival). All rats were provided food and water ad libitum and were housed on a 12:12 light/dark cycle. After arrival, animals were allowed 1 week to habituate to the facility and an additional week of handling prior to any experimentation. Rats used for electrophoretic mobility shift assay (EMSA) and quantitative polymerase chain reaction (qPCR) experiments were group housed, and rats used for the sucrose preference experiment were singly housed. All procedures were approved by the Institutional Animal Care and Use Committee and were in accordance with NIH guidelines.

### L822429 and lipopolysaccharide treatment

The NK1R antagonist L822429 was dissolved in 2-hydroxypropyl-β-cyclodextrin (45% *w*/*v*) and injected i.p. at a dose of 30 mg/kg in a volume of 2 ml/kg. L822429 treatment was administered 1 h prior to LPS treatment. LPS (strain 0111:B4, Sigma) was dissolved in sterile 0.9% saline and injected i.p. at a dose of 200 μg/kg in a volume of 1 ml/kg. In EMSA and qPCR experiments, rats were sacrificed by live decapitation 2 h after injection. Brains were extracted, hippocampal tissue was dissected, and samples were snap frozen in isopentane. Samples were stored at − 80 °C until tissue processing.

### Electrophoretic mobility shift assay

To investigate the role of the NK1R in LPS-induced activation of NFkB-DNA-binding potential, rats (*n* = 6/group) were treated systemically with vehicle or L822429 followed by injection of saline or LPS. Nuclear protein extracts were prepared using Active Motif Nuclear Extract Kit (Active Motif), and protein concentration in each sample was determined using Pierce™ BCA Protein Assay Kit (Thermo Fisher Scientific). For EMSA, nuclear protein samples (15 μg) were incubated with Odyssey® EMSA Buffer Kit (LI-COR) and NFkB IRDye® 700 infrared dye-labeled oligonucleotide. Loading dye was added to each sample, and the gel (6% DNA retardation gel, Invitrogen, Life Technologies) was run at 200 V at 4 °C. Gels were imaged, and band intensity was quantified using Li-Cor Odyssey CLx Infrared Imaging System.

### Quantitative polymerase chain reaction

The expression of proinflammatory cytokines IL1β, IL6, and TNFα is known to be strongly activated by NFkB. To evaluate the role of the NK1R in LPS-induced increases in proinflammatory cytokine expression, rats (*n* = 5–6/group) were treated systemically with vehicle or L822429 followed by injection of saline or LPS. Hippocampal samples were homogenized, and RNA was extracted using PureLink™ RNA Mini Kit (Ambion, Life Technologies) according to the manufacturer’s instructions. We chose to analyze cytokine mRNA levels within the hippocampus based on previous work demonstrating a significant effect of LPS treatment on proinflammatory cytokine expression in this region [36, 37]*.* Samples were then reversed transcribed using SuperScript® III Kit (Invitrogen, Thermo Fisher Scientific) according to the manufacturer’s instructions. qPCR was performed on each sample in triplicate using the following Taqman primers: TNF-α (Rn0152859_g1), IL-6 (Rn0140330_m1), IL-1β (Rn0058432_m1), and GAPDH for internal control (Rn01775763_g1; Life Technologies). qPCR analysis was performed on a Life Technologies QuantStudio6 machine.

### Sucrose preference test

To assess the effect of NK1R antagonism on LPS-induced anhedonic-like behavior, a sucrose preference test was conducted [38–40]. Rats (*n* = 6/group) were provided with one bottle containing 1% (*w*/*v*) sucrose solution and one bottle containing water for 2 days to establish a baseline sucrose preference. Bottle position was switched every 24 h to prevent development of a side preference. Sucrose solution was then removed so that only water was available, and rats were treated with L822429 or vehicle, followed by saline or LPS. The 1% sucrose solution was reintroduced 12 h after LPS treatment, and water and sucrose intake was measured 12 h later. Baseline and post-treatment sucrose preference was expressed as proportion of fluid intake that was accounted for by consumption from the sucrose bottle. For example, a value of 0.8 would indicate that 80% of the total fluid consumption was from the bottle containing the sucrose solution.

## Results

### NK1R antagonism attenuates LPS-induced increase in NFkB-DNA-binding potential in the hippocampus

**Fig. 1:**
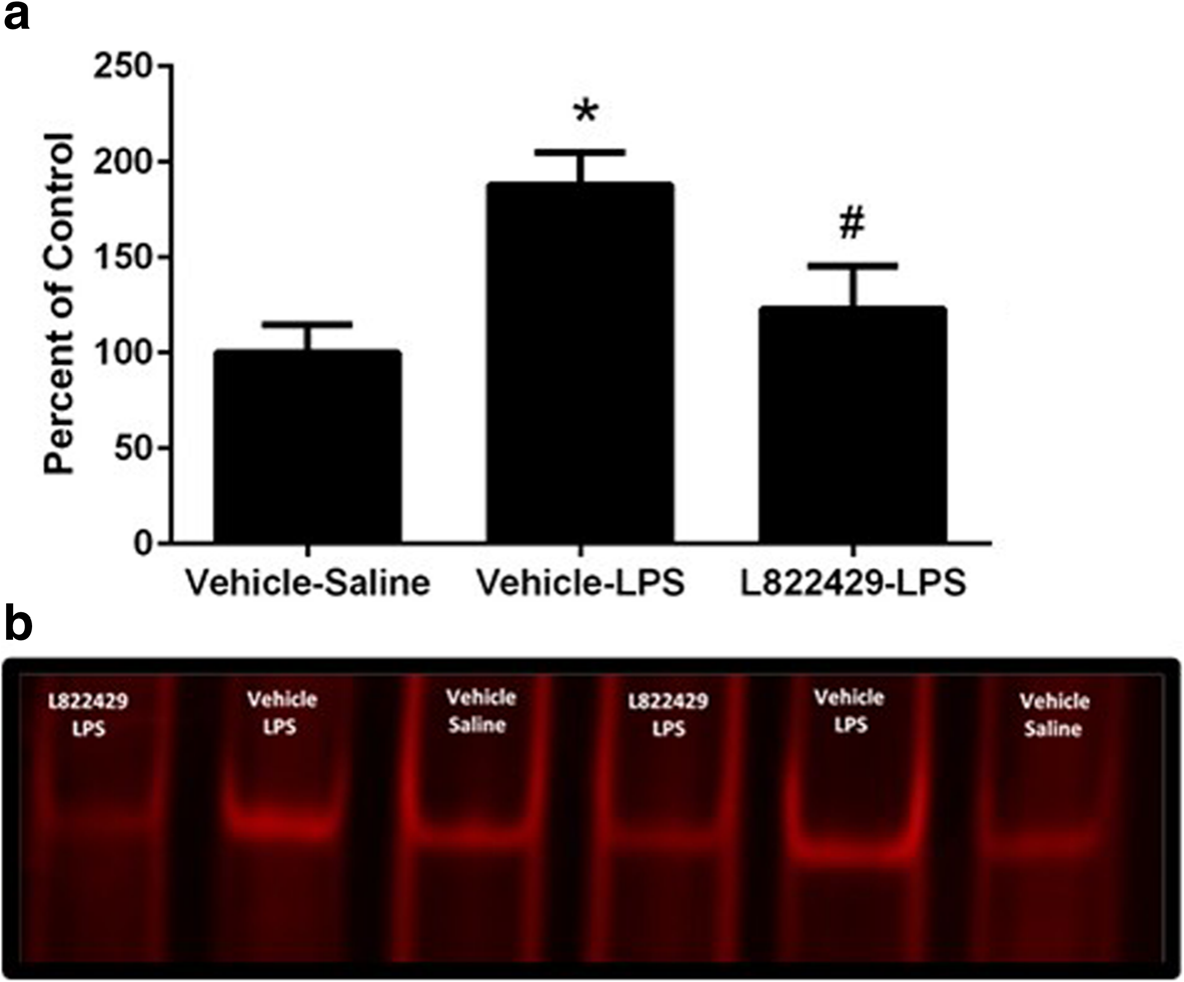
Effect of neurokinin-1 (NK1R) antagonism on LPS-induced increase in NFkB-DNA-binding activity in the hippocampus. Rats were treated with L822429 (30 mg/kg) 1 h prior to LPS (200 μg/kg) and sacrificed 2 h after LPS injection. **a** Electrophoretic mobility shift assay (EMSA) quantification: data expressed as percent of vehicle-saline control. **b** Representative image of EMSA results. *n* = 6/group. *p < 0.05, compared to the vehicle-saline, ^#^*p* < 0.05 compared to the vehicle-LPS

One-way ANOVA indicated that optical density of shift bands in the EMSA assay were significantly different between treatment groups (Fig. 1a, *F*_2,15_ = 6.158, *p* < 0.05). Representative image shown in Fig. 1b. Post hoc analysis using Newman-Keuls tests revealed that vehicle-LPS-treated subjects had significantly higher intensity when compared to both vehicle-saline-treated subjects and L822429-LPS treated subjects (*p* < 0.05 for both comparisons). Intensity in the L822429-LPS-treated group was not significantly different from the vehicle-saline-treated group. This suggests that LPS induced an increase in NFkB translocation to the nucleus and NFkB-DNA-binding potential that was attenuated by pretreatment with the NK1R antagonist.

### NK1R antagonism attenuates LPS-induced increase in proinflammatory cytokine expression in the hippocampus

**Fig. 2:**
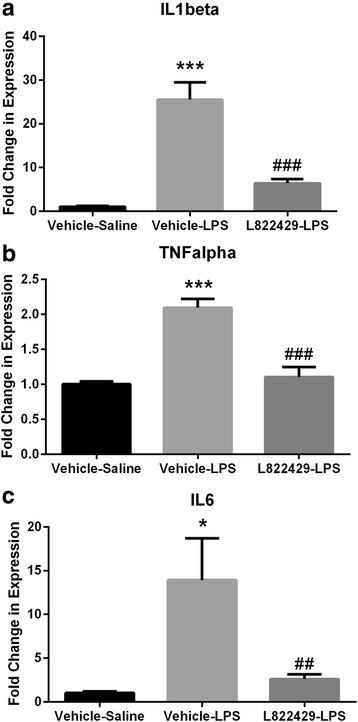
Effect of neurokinin-1 receptor (NK1R) antagonism on LPS-induced increase in proinflammatory cytokine expression in the hippocampus. Rats were treated with L822429 (30 mg/kg) 1 h prior to LPS (200 μg/kg) and sacrificed 2 h after LPS injection. mRNA transcript levels were measured using qPCR. NK1R antagonism attenuates LPS-induced increase in expression of 1L1-β (**a**)**,** TNF-α (**b**), and IL-6 (**c**). *n* = 5–6/group. *p < 0.05, ***p < 0.001, compared to the vehicle-saline group. ^##^*p* < 0.01, ^###^*p* < 0.001, compared to the vehicle-LPS group

One-way ANOVA revealed that IL-1β, TNF-α, and IL-6 mRNA levels were significantly different between treatment groups (Fig. 2a: *F*_2,13_ = 31.89, *p* < 0.0001; Fig. 2b: *F*_2,13_ = 25.29, *p* < 0.0001; Fig. 2c: *F*_2,13_ = 7.048, *p* < 0.01, respectively). Post hoc analysis using Newman-Keuls tests revealed that the vehicle-LPS-treated group had significantly higher expression of IL-1β, TNF-α, and IL-6 when compared to the vehicle-saline-treated group (*p* < 0.001, *p* < 0.001, and *p* < 0.05, respectively) and when compared to the L822429-LPS-treated group (*p* < 0.001, *p* < 0.001, and *p* < 0.01, respectively). IL-1β, TNF-α, and IL-6 levels were not significantly different between L822429-LPS-treated and vehicle-saline-treated groups. These results indicate that LPS induces increased expression of proinflammatory cytokines in the hippocampus and this is attenuated by NK1R antagonism.

### NK1R antagonism attenuates LPS-induced anhedonia

**Fig. 3:**
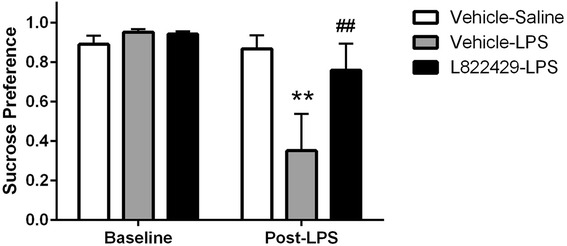
Effect of neurokinin-1 (NK1R) antagonism on LPS-induced anhedonia. Rats were treated with L822429 (30 mg/kg) 1 h prior to LPS (200 μg/kg) and sucrose consumption began 12 h after LPS injection. Systemic treatment with 200 μg/kg LPS decreases voluntary sucrose consumption in the two-bottle choice test. NK1R antagonism with systemic administration of 30 mg/kg L822429 attenuates LPS-induced decrease in sucrose preference. Data shown was collected 12 h post-LPS treatment. *n* = 6/group. **p < 0.01, compared to the vehicle-saline group, ^##^*p* < 0.01 compared to the vehicle-LPS group

Two-way ANOVA revealed a significant effect of test phase (baseline versus post-treatment; *F*_1,15_ = 11.03, *p* < 0.01) and a significant interaction between the factors of test phase and treatment (*F*_2,15_ = 4.512, *p* < 0.05). Post hoc analysis using Newman-Keuls tests revealed that during the baseline phase, the average sucrose preference did not differ between groups. However, the vehicle-LPS-treated group had significantly decreased sucrose preference post-treatment when compared to both the vehicle-saline-treated group and the L822429-LPS-treated group (Fig. 3, *p* < 0.01 for both comparisons). Sucrose preference post-treatment in the L822429-LPS-treated group and the vehicle-saline-treated group were not significantly different. These data suggest that NK1R activation is, at least in part, required for LPS-induced suppression of sucrose preference.

## Discussion

In this study, we demonstrate that the cellular and behavioral effects of LPS administration are in part dependent upon NK1R signaling. First, we show that NFkB-DNA-binding potential and subsequent expression of proinflammatory cytokines by systemic LPS injection is attenuated by systemic injection of the NK1R antagonist L822429. We show these effects in ex vivo brain tissue homogenates from the hippocampus. In previous studies, the role of NK1R in LPS-induced cellular effects has been demonstrated in vitro or in non-neural tissue; here, we extend those findings to show that this mechanism functions in the nervous system in vivo. Furthermore, many in vivo studies examining the impact of LPS on NFkB activation use the endpoint measure of cytokine expression. Here, we use this measure as well but also demonstrate that NFkB binding potential is increased by systemic LPS treatment. This is important because NFkB is unlikely to be the only transcription factor that influences the expression of proinflammatory genes. Additionally, we show that the anhedonia-inducing effects of LPS administration are also influenced by the NK1R. Thus, both the cellular and behavioral effects of LPS injection depend, at least in part, upon the action of the NK1R.

In this study, we assessed the effect of systemic administration of LPS on NFkB/cytokine activity in the hippocampus. However, it is unlikely that LPS injected through the intraperitoneal route reaches the brain. Thus, there is likely to be an intermediate signaling process that induces the CNS effect that we observe. Alternatively, NK1R actions in the periphery could influence the amount of peripheral cytokine release that is induced following LPS injection. Future experiments will examine these specific routes by which the NK1R may influence LPS effects.

Here, we show that LPS-induced anhedonia is influenced by NK1R activation. In addition to this anhedonia effect, LPS exposure has been shown to influence other behaviors of interest including sensitivity to painful stimuli [41], alcohol intake [42], and anxiety [43, 44]. Future studies will examine the role of the NK1R in additional LPS-induced behaviors. This is of particular interest because there is a considerable overlap between the types of behaviors that are influenced by NK1R signaling and those that are induced by LPS.

## Conclusions

Taken together, the results presented here suggest that the NK1R contributes to multiple aspects of LPS challenge. The role of the NK1R may also extend to neuroinflammatory responses in general, and this receptor may thus influence psychiatric conditions that are mediated by chronic inflammation including depression, chronic pain, alcohol dependence, and others. FDA-approved NK1R antagonists are currently on the market; aprepitant has been used for several years to treat chemotherapy-induced nausea. Therefore, the efficacy of this drug could be tested in clinical trials for psychiatric conditions mediated by inflammation. While past clinical trials using NK1R antagonists have produced mixed results, negative findings have possibly been influenced by complex factors including drug dosing and pharmacogenetic interactions (see [45, 46] for discussion of this topic).

## Abbreviations

EMSA: Electrophoretic mobility shift assay
IL-1β: Interleukin-1β
LPS: Lipopolysaccharide
NFkB: Nuclear factor kappa light-chain-enhancer of activated B cells
NK1R: Neurokinin-1 receptor
qPCR: Quantitative polymerase chain reaction
SP: Substance P
TNF-α: Tumor necrosis factor-α
